# Human Mesenchymal Stromal Cells Enhance Cartilage Healing in a Murine Joint Surface Injury Model

**DOI:** 10.3390/cells10081999

**Published:** 2021-08-06

**Authors:** Jade Perry, Anke J. Roelofs, Claire Mennan, Helen S. McCarthy, Alison Richmond, Susan M. Clark, Anna H. K. Riemen, Karina Wright, Cosimo De Bari, Sally Roberts

**Affiliations:** 1 The Robert Jones & Agnes Hunt Orthopaedic Hospital NHS Foundation Trust, Oswestry, Shropshire SY10 7AG, UK; claire.mennan@nhs.net (C.M.); helen.mccarthy6@nhs.net (H.S.M.); karina.wright1@nhs.net (K.W.); sally.roberts4@nhs.net (S.R.); 2 The School of Pharmacy & Bioengineering, Keele University, Staffordshire ST5 5BG, UK; 3 The Tissue Engineering & Regenerative Therapies Centre versus Arthritis, Cambridge CB2 2QQ, UK; 4 Arthritis and Regenerative Medicine Laboratory, Institute of Medical Sciences, University of Aberdeen, Aberdeen AB25 2ZD, UK; a.roelofs@abdn.ac.uk (A.J.R.); a.richmond@abdn.ac.uk (A.R.); susan.clark@abdn.ac.uk (S.M.C.); ariemen@abdn.ac.uk (A.H.K.R.); c.debari@abdn.ac.uk (C.D.B.)

**Keywords:** mesenchymal stromal cells, umbilical cord, bone marrow, cartilage repair, allogeneic cell therapy, osteoarthritis, mouse models

## Abstract

Human umbilical cord (hUC)- or bone marrow (hBM)-derived mesenchymal stromal cells (MSCs) were evaluated as an allogeneic source of cells for cartilage repair. We aimed to determine if they could enhance healing of chondral defects with or without the recruitment of endogenous cells. hMSCs were applied into a focal joint surface injury in knees of adult mice expressing tdTomato fluorescent protein in cells descending from Gdf5-expressing embryonic joint interzone cells. Three experimental groups were used: (i) hUC-MSCs, (ii) hBM-MSCs and (iii) PBS (vehicle) without cells. Cartilage repair was assessed after 8 weeks and tdTomato-expressing cells were detected by immunostaining. Plasma levels of pro-inflammatory mediators and other markers were measured by electrochemiluminescence. Both hUC-MSC (*n* = 14, *p* = 0.009) and hBM-MSC (*n* = 13, *p* = 0.006) treatment groups had significantly improved cartilage repair compared to controls (*n* = 18). While hMSCs were not detectable in the repair tissue at 8 weeks post-implantation, increased endogenous Gdf5-lineage cells were detected in repair tissue of hUC-MSC-treated mice. This xenogeneic study indicates that hMSCs enhance intrinsic cartilage repair mechanisms in mice. Hence, hMSCs, particularly the more proliferative hUC-MSCs, could represent an attractive allogeneic cell population for treating patients with chondral defects and perhaps prevent the onset and progression of osteoarthritis.

## 1. Introduction

Joint surface defects (JSD), whether they are focal chondral or osteochondral defects, are observed in more than half of all arthroscopic procedures in the knee and are associated with pain and cartilage loss, which can contribute to the pathogenesis of osteoarthritis (OA) [1,2]. JSDs can be as debilitating for patients as anterior cruciate ligament injury or end-stage OA [3]. Consequently, joint surface restoration is of great importance in modern medicine.

The cell therapy treatment, autologous chondrocyte implantation (ACI), has been shown to be a successful treatment option of chondral lesions, even in young individuals with early OA, for whom there are few other treatment options [4]. Minas et al. (2010) found 92% of 153 patients with a mean age of 38.3 years to be functioning well 5 years post-ACI [4], delaying the need for arthroplasty, which is an important outcome for individuals of this age, with lower implant survival in young active patients [5]. However, whilst clinically successful, and deemed to be cost-effective by the UK National Institute for Health and Care Excellence (NICE) for treating symptomatic cartilage defects in the knee (www.nice.org.uk/ta477, accessed on 10 June 2021), the use of ACI is not without its challenges. Being an autologous cell therapy, it requires two surgical procedures, one for harvesting the chondrocytes, which in itself can cause some short-term joint morbidity [6], and the second for implanting the cells [7]. This incurs logistical and financial costs. In addition, the manufacturing cost for an autologous product is inevitably much greater than for each treatment batch of an allogeneic cell therapy product.

Mesenchymal stromal cells (MSCs) are increasingly being used as an alternative cell therapy for treating cartilage defects or OA in the knee [8]. In our centre, we have been investigating human umbilical cord-derived MSCs (hUC-MSCs) and have shown them to have similar trilineage differentiation capability, CD-immunoprofiles and immunomodulatory ability as human bone marrow (hBM)-derived MSCs, while having a greater proliferative capacity [9,10]. While the exact mode of action (MoA) of MSCs remains unknown, an increasing body of evidence suggests that MSCs exhibit trophic effects on endogenous cell populations, through the secretion of numerous soluble factors, such as growth factors and cytokines, as well as through the production of immunomodulatory and anti-inflammatory molecules, in addition to extracellular vesicles (EVs) [11]. Previous in vitro work in our centre has shown that indoleamine 2,3-dioxygenase (IDO), a potent immunomodulatory molecule, is upregulated by both hBM-MSCs and hUC-MSCs following exposure to a pro-inflammatory stimulus (IFN-γ) [12]. IDO appears to minimise the local inflammatory response through the depletion of tryptophan via the kynurenine pathway, causing the suppression of T-cells [13].

MSCs therefore appear to have potential as a source of allogeneic cells for cartilage repair. An in vivo study of hUC-MSCs injected into a murine model of established OA demonstrated no or limited ability to restrict or reverse severe osteoarthritic changes, but did not appear to elicit any inflammatory reaction in this xenogeneic study [14]. In the present study, a murine model of joint surface injury (more akin to the clinical situation which ACI was designed to treat) was utilised [15,16] and treated with hUC-MSCs or hBM-MSCs, which were culture expanded in a bioreactor [12].

## 2. Materials and Methods

### 2.1. Human Samples

All samples of human umbilical cords were obtained after patients had provided written informed consent; favourable ethical approval was given by the National Research Ethics Service (10/H1013/62). Umbilical cords (*n* = 3) were obtained from natural births from mothers aged 23, 24 and 35 years; MSCs were isolated from the tissue enzymatically and culture expanded via a hybrid process [12]. They were cultured for a single passage using standard tissue culture techniques in Dulbecco’s Modified Eagle’s Medium (DMEM/F12, Life Sciences, Paisley, UK) containing 1% (*v*/*v*) penicillin and streptomycin (P/S, Life Sciences, Paisley, UK) and 10% (*v*/*v*) foetal calf serum (FCS; Life Sciences, Paisley, UK), hereafter referred to as complete culture medium [12]. Cells were then harvested and 5 million seeded into the Quantum^®^ bioreactor (Terumo BCT Inc, Lakewood, CO, USA) for further individual culture expansion in complete culture medium [12]. Human bone marrow aspirate samples (*n* = 3; purchased from Lonza (Walkersville, MD, USA) from males aged 22, 26 and 32 years), were also cultured individually in the Quantum^®^ bioreactor in complete culture medium. BM aspirates were culture-expanded by inserting 20 mL of BM for one passage after which 10 million cells were reseeded into the Quantum^®^ to undergo a second passage, before being harvested and characterised [12]. All MSC populations were assessed for the presence of CD19, CD34, CD45, Human Leukocyte Antigen (HLA)-DR, CD73, CD90, CD105 (International Society for Cellular Therapy (ISCT) MSC markers) [17], CD271, Receptor Tyrosine Kinase-like Orphan Receptor 2 (ROR2), Fibroblast Growth Factor Receptor 3 (FGFR3) (chondrogenic/MSC markers), CD151, CD39, CD44, CD49, CD163, CD166 (chondrogenic markers) and CD106 and CD317 (immunomodulatory markers) by flow cytometry with appropriate isotype-matched IgG negative controls as reported previously [12].

Conditioned medium (CM) was collected from Quantum^®^ expanded hUC-MSCs following a subsequent passage in standard tissue culture. During this additional passage, cells were left unstimulated or exposed to either 25 ng/mL IFN-γ or an “inflammatory cocktail” (containing 25 ng/mL IFN-γ, 10 ng/mL interleukin (IL)-1β and 50 ng/mL TNF-α) for a 24 h period. The CM was analysed using a multiplex electrochemiluminescence assay (ECL, from MesoScale Diagnostics (MSD; Rockville, MD, USA)) for the following analytes: granulocyte-macrophage colony-stimulating factor (GM-CSF), IL-1 receptor antagonist (RA), IL-4, IL-6, IL-8, IL-10, interferon γ-induced protein (IP)-10, macrophage chemotactic protein (MCP)-1, vascular endothelial growth factor (VEGF) and stromal cell-derived factor (SDF)-1α.

### 2.2. Mice and In Vivo Procedures

All animal experimental protocols were approved by the UK Home Office and the Animal Welfare and Ethical Review Committee of the University of Aberdeen, and are reported in compliance with the Animal Research: Reporting of In Vivo Experiments (ARRIVE) guidelines. Tg(Gdf5-cre-ALPP)1Kng mice, or Gdf5-Cre in short [18], were provided by David Kingsley (Stanford, CA, USA) and maintained on an FVB background. Cre-inducible tdTomato mice, B6.Cg-Gt(ROSA)26Sortm14(CAG-tdTomato)Hze/J, or Tom in short [19], were obtained from JAX (stock number 6774) and maintained on a C57Bl/6 background. Mice were cross-bred and resulting female double-hemizygous Gdf5-Cre;Tom offspring were used in this study. The tdTomato (Tom) reporter allows identification (via its red fluorescent protein) of Gdf5-lineage cells descending from the embryonic joint interzone as reported [16]. ‘Leaky’ mice were excluded a priori based on analysis of Tom expression in blood [16]. Mice were bred at Charles River Laboratories, UK and shipped to the animal research facility in Aberdeen between 9 and 18 days before surgery. Mice were group-housed with 12-h light/dark cycles, and fed ad libitum water and food.

At 8-to-10 weeks of age, a full-thickness joint surface injury (JSI) was performed by medial parapatellar arthrotomy as previously described [15]. Mice (*n* = 54) were anaesthetized with ketamine (50 mg/kg) and medetomidine (0.67 mg/kg) and given atipamezole (1 mg/kg) post-operatively. Using a dissecting microscope, an incision was made to open up the skin over the knee, followed by an incision along the medial side of the patellar ligament and through the quadriceps muscle to aid patellar dislocation. The patellar groove was exposed and a longitudinal full-thickness cartilage defect along the length of the groove was made using a 25-gauge needle. Human MSCs, or vehicle, were then applied as detailed below, before the patella was re-located and incisions were closed by suturing [16].

**Figure 1:**
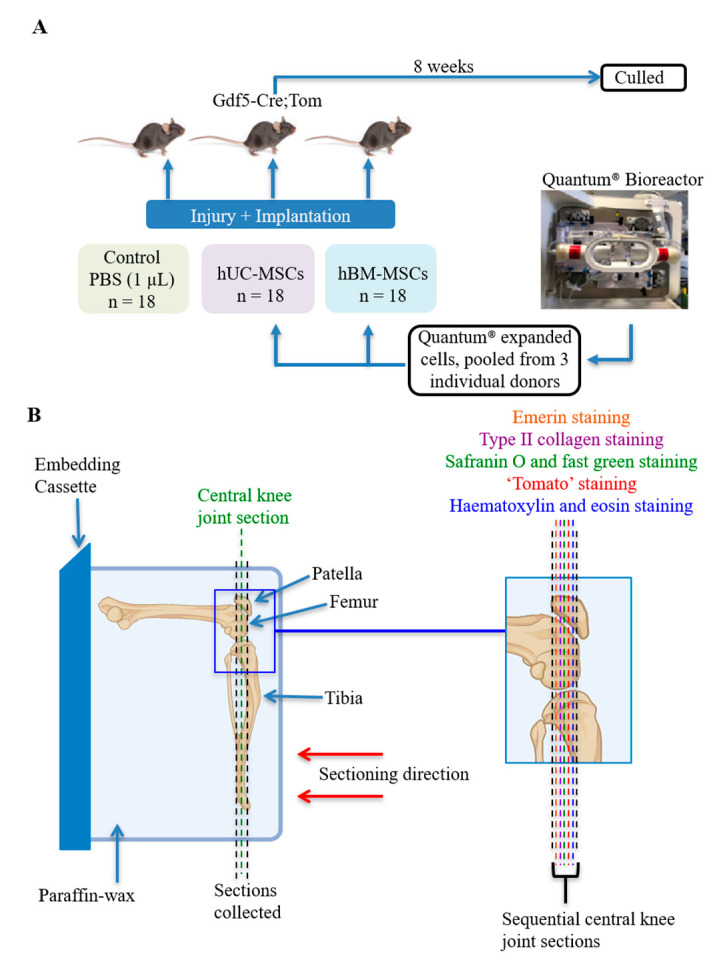
A schematic to show the joint surface injury (JSI) experimental design and histological staining process (partially created using Biorender.com (accessed on 15 May 2021)). (**A**) At the time of injury, Quantum-expanded human BM-MSCs or human UC-MSCs (2 × 10^5^ cells in 1 µL of PBS) were implanted, unless the mice were in the vehicle control group, in which case they received PBS alone. The mice were culled after 8 weeks. (**B**) The orientation of paraffin-embedded murine knee joints in the JSI model. The knee joint sections were cut as indicated, with sequential central knee joint sections being stained with safranin O and fast green, haematoxylin and eosin, or antibodies against type II collagen, emerin or tdTomato.

Quantum-expanded hUC-MSCs (3 donors) or hBM-MSCs (3 donors) were defrosted from liquid nitrogen storage, cultured for up to 3 passages in regular cell culture flasks in complete culture medium, then harvested by trypsinisation on the day of surgery. Cells from the 3 donors were pooled together, washed twice in phosphate buffered saline (PBS), and resuspended in PBS at 2 × 10^5^ cells/µL and kept on ice until implantation. To control for the effects of any traces of culture media in the prepared cell suspensions, the vehicle was prepared by processing culture media without cells the same way. The hUC-MSCs, hBM-MSCs, or vehicle only, were applied to the cartilage defect at the time of surgery (18 mice/group) by dropping 2 × 10^5^ cells, or 1 µL vehicle only, onto the defect using a Hamilton syringe (Hamilton, Switzerland) before closing the joint (Figure 1). Required sample size was estimated based on previous data of histological outcome of repair in this model as determined by the Wakitani grading system [16,20] which was the primary outcome measure in this study. Surgeries were performed over 4 days, with a similar number of mice in each treatment group on each day. To minimise the time between cell harvest and implantation, on each day cages of mice were randomly assigned to one of the treatment groups with no specific randomisation process applied and underwent surgery sequentially. The order of treatment groups varied between surgery days to control for any influence of sequence. All mice were female, between 59 and 70 days old at the time of surgery (average age 63–65 days in all groups) and continued to be housed in similar sized groups of 4–6 mice per cage in the same room. Researchers conducting the in vivo procedures were aware of treatment allocation, as it was not possible to perform this blinded due to the presence of cells being visible in the high-cell-density suspension at time of implantation. Eight weeks later, blood was collected by cardiac puncture under terminal anaesthesia followed by cervical dislocation, and the operated knee joints were harvested for histological analysis. One mouse receiving hUC-MSCs developed redness and swelling of the knee 2 days after surgery and was culled. Histology confirmed inflammatory infiltrates in the knee. Two mice receiving hBM-MSCs died within 24 h of surgery, with exact cause of death undetermined. Numbers of murine knee joints available for study were reduced further due to technical issues encountered during sectioning (*n* = 4). In addition, mice with patellar dislocation (*n* = 2), a known complication of this type of surgery in a small percentage of mice which interferes with normal cartilage repair processes [21], were excluded from analysis, resulting in the following number of mice in each group: vehicle (*n* = 18), hUC-MSCs (*n* = 14) and hBM-MSCs (*n* = 13).

### 2.3. Histology, Immunohistochemistry and Electrochemiluminescence

The intact knee joint specimens were fixed in 4% formaldehyde (Sigma-Aldrich, Dorset, UK) in PBS overnight at 4 °C, decalcified in 4% EDTA for approximately 2 weeks before transferring to 70% ethanol. Flexed knee joints were then embedded in paraffin wax and sectioned (5 µm thick) according to Figure 1. Sections were stained with haematoxylin and eosin (H&E) or safranin O and fast green (SO/FG). Cartilage repair and synovitis were assessed in each knee joint by 3 fully blinded independent scorers (J.P, H.S.M and C.M), who were not involved in the in vivo procedures. The Wakitani grading system was used to assess cartilage repair in the injury site on three central SO/FG-stained repair sections, spanning approximately 100 μm, with a score of 0 (normal cartilage) to 14 (absence of repair) [20]. Synovitis was assessed on a single central H&E-stained section using a modified Jackson score [22], with a 10-point system (0 being normal to 10 being inflamed), evaluating the severity of synovial hyperplasia, sub-synovial stroma, sub-synovial inflammation and synovial exudate (Appendix A, Table A1) [22,23]. Synovitis was assessed on the patellar compartment on the lateral side of the joint only, to prevent any confounding influence of the injury caused by the medial parapatellar arthrotomy. When any score varied by 2 points or more, the knee joint was re-assessed as a collective by all three scorers and a consensus reached. Data were only unblinded to the different treatment groups after all analyses were completed.

Tissue sections were also investigated, fully blinded, via immunohistochemical staining for the presence of type II collagen (with monoclonal antibody CIICI (1:50); Developmental Studies Hybridoma Bank (DSHB), Iowa City, IA, USA) and tdTomato-positive cells (Tom+; with polyclonal rabbit anti-red fluorescent protein (RFP) antibody (1:200); Rockland Immunochemicals Inc, Limerick, Republic of Ireland). Sections were deparaffinised in xylene and rehydrated in an ethanol series (100–50%). For antigen retrieval, sections were treated with 0.2N HCl for 10 min, digested in 5 mg/mL pepsin (Sigma-Aldrich, Dorset, UK) in 0.2N HCl for 2 h at 37 °C and then washed in distilled water. Immunolocalisation of Tom+ cells and type II collagen was performed as previously described, with diaminobenzadine as the chromagen [6,24]. Negative controls were performed on adjacent sections using an isotype-matched mIgG2a antibody (Dako, Glostrup, Denmark) for type II collagen and rabbit serum (Abcam, Cambridge, UK) for Tom+ staining, in place of the primary antibodies.

Immunofluorescence (IF) was used to determine if human UC-MSCs could be identified in the murine knee joint, with mouse monoclonal antibody, MANEM1, clone 5D10, against the epitope peculiar to human emerin which is not present in murine species (kindly provided by Dr Heidi Fuller (Oswestry)) [14,25]. All steps were performed at room temperature and sections were washed three times in 0.2% Triton X-100 in PBS between steps unless otherwise stated. Following deparaffinisation and rehydration, antigen retrieval was performed by boiling in sodium citrate buffer, pH 6 for 20 min. Sections were then blocked for 30 min in IF blocking buffer (1% bovine serum albumin/10% horse serum/10% FCS in PBS). Sections were blotted and incubated with the primary antibody (MANEM1, diluted 1:10 in IF blocking buffer) for 2 h. Adjacent sections were stained with an isotype-matched IgG1 (Dako, Glostrup, Denmark) as a negative control. Sections were then incubated with the secondary antibody, goat anti-mouse IgG Alexa Fluor 488 (diluted 1:400 in IF blocking buffer, Paisley, Life Technologies, UK) for 1 h, before washing and mounting the slides in hard-set medium with DAPI (VECTASHIELD^®^, Vector Laboratories, Burlingame, CA, USA). Images were obtained using a Leica SP5 confocal microscope.

The quantity of tomato and emerin-positive cells in the repair region was manually assessed and data were expressed as the percentage of cells that stained positive in the repair region. To ensure fair analysis of the presence of Tom+ cells, mice without repair in the JSI were excluded. To analyse the distribution of type II collagen in the repair region, positive extracellular matrix staining was recorded as a percentage of the total area of the joint surface injury, using image analysis software, NIS-Elements: Version 3.2, Nikon, Surrey, UK.

Plasma samples obtained after culling were analysed, fully blinded, for potential indicators of inflammation using a custom-designed murine multiplex ECL (from MSD) for the following analytes: GM-CSF, IL-1β, IL-4, IL-6, IL-10, TNF-α, IFN- γ, MCP-1 and VEGF. The presence of blood staining was also assessed using a visual score [26] ranging from 0 (no haemolysis) to 3 (severe).

### 2.4. Statistics

All data were assessed using non-parametric analyses. Differences between treatment groups (e.g., no cells, hBM-MSCs and hUC-MSCs for histology and inflammatory markers) were evaluated using a Kruskal–Wallis test with Dunn’s corrections. Data were expressed as the mean ±95% confidence intervals (CI), with a *p* value of <0.05 deemed statistically significant, using Prism (version 8.1.2, GraphPad Software, La Jolla, CA, USA). The inter-rater reliability of the Wakitani score and synovitis score, by three raters (J.P, H.S.M and C.M) was determined using the two-way intra-class correlation coefficient for agreement (ICC(A,1)) as calculated with R vs. 3.6.0 (R Foundation for Statistical Computing, Vienna, Austria) using the package “irr” [27], together with the intra-rater reliability by the individual raters who scored 5 murine knee joints, 10 times each [28]. The influence of haemolysis in the plasma samples was assessed using the Jonckheere-Terpstra test [29,30].

## 3. Results

### 3.1. Histological and Immunohistochemical Analyses of Joint Changes after JSI

**Figure 2:**
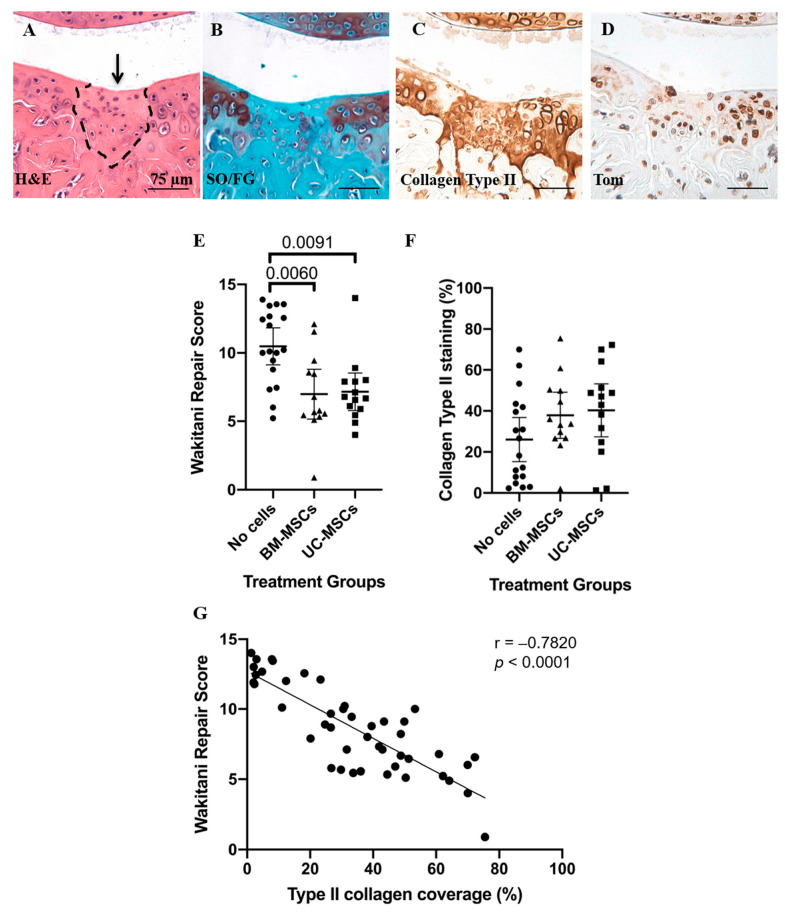
Adjacent sections from one mouse used for histology and immunohistochemistry showing (**A**) the repair tissue (dashed line, H&E staining) which has formed 8 weeks post-injury in mice that received human UC-MSCs (arrow), with (**B**) less staining for safranin O but (**C**) significant collagen type II immunostaining, compared to the adjacent native cartilage. (**D**) Cells populating the repaired region were endogenous cells that descended from the cells of the embryonic joint interzone, as shown by positive staining for tdTomato. Scale bars = 75 µm. (**E**) Assessment of cartilage repair tissue. The Wakitani cartilage repair score was significantly lower for both human BM-MSCs and human UC-MSCs, indicating better repair compared with the no cell control. (**F**) Immunohistochemistry demonstrated that there was no significant difference between any of the treatment groups with regard to the amount of collagen type II staining in the repair tissue. Data are presented as the mean ±95% CI. (**G**) Area of repair tissue immunostaining positive for type II collagen plotted against the Wakitani scores for all knee joints from all treatment and control groups.

**Figure 3:**
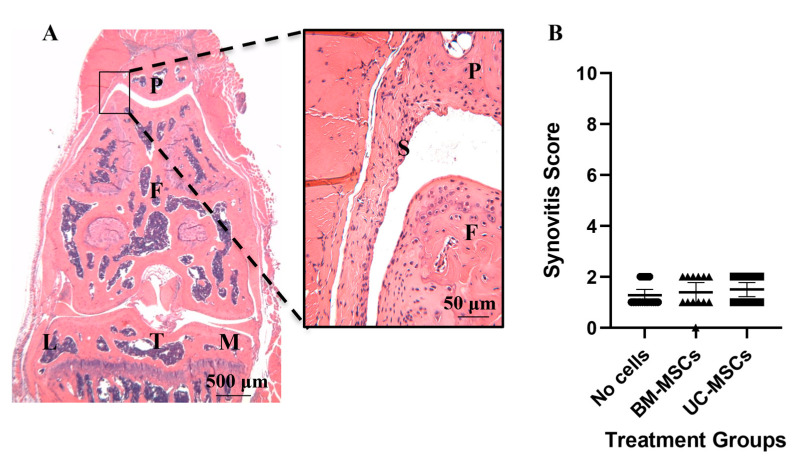
Assessment of synovial reaction to the JSI following treatment. A representative H&E stained section of (**A**) a murine knee joint 8 weeks post-injury from a mouse that received hUC-MSCs. The boxed region, where synovitis was scored, is also shown at a higher magnification. Synovitis was assessed using a 10-point system (0 being normal to 10 being severely inflamed). Synovium, S; patella, P; femur, F; tibia, T; lateral side, L; medial side, M. (**B**) Synovial reaction was not significantly different between any of the treatment groups. Data are presented as the mean ±95% CI.

**Figure A1:**
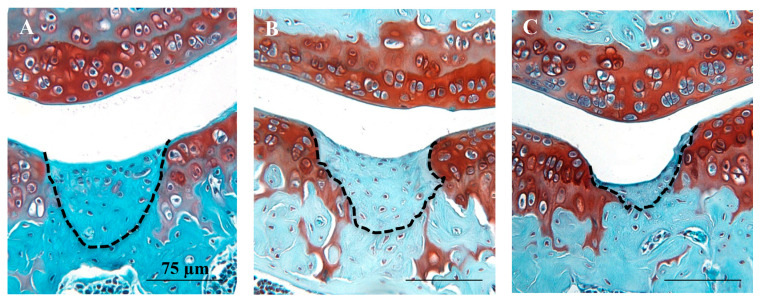
Cartilage formation and repair after joint surface injury. Safranin O and fast green stained sections from individual mouse knees showing the injury site in the patellar groove at 8 weeks post-injury in the sample groups (**A**) human UC-MSCs, (**B**) human BM-MSCs and (**C**) the control group (no cells injected). Clear repair tissue (indicated by dashed lines) can be seen in the MSC treated sections, whereas only minimal repair is present in the control group. Scale bars = 75 μm.

Analysis of cartilage repair in safranin O and fast green stained sections showed that both hMSC treatment groups had significantly lower mean Wakitani scores (i.e., better repair) compared to the vehicle group (*p* = 0.009 for hUC-MSCs and *p* = 0.006 for hBM-MSCs) (Figure 2 and Appendix A Figure A1). Furthermore, there was a low level of synovial hyperplasia but no overt signs of synovitis and no significant differences in synovitis scores between any of the treatment groups (no cells vs. hBM-MSCs *p* > 0.999; no cells vs. hUC-MSCs *p* = 0.826; hBM-MSCs vs. hUC-MSCs *p* > 0.999) (Figure 3). The inter-rater reliability (ICC) between scorers of the Wakitani cartilage repair score and synovitis score was 0.98 and 0.88, respectively and the intra-rater class reliability was 0.96 and 0.78.

**Figure 4:**
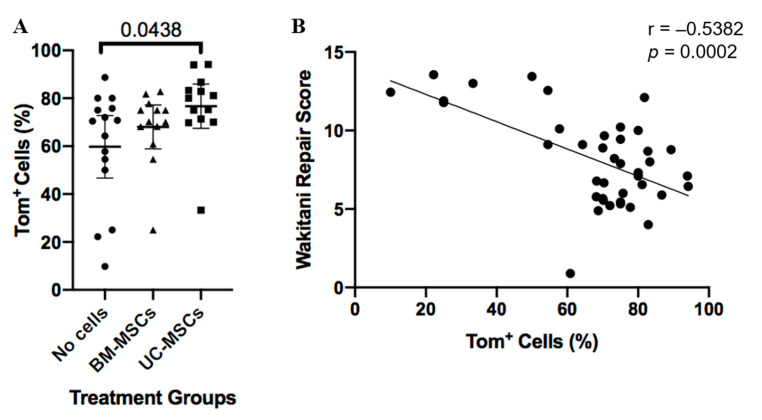
Tomato positive (Tom+) cells in repair tissue. (**A**) Cell-treated groups had more tomato-positive cells compared to the no cell control group, but this was only significant for human UC-MSCs. This indicated that a greater number of cells populating the repaired region originated from the murine joint interzone, as shown by Tom+ staining. Data are presented as the mean ±95% CI. (**B**) Percentage of Tom+ cells within the repair tissue plotted against the Wakitani scores for all knee joints from all treatment and control groups.

Immunohistochemistry demonstrated the presence of Tom+ Gdf5-lineage cells in articular cartilage, ligaments, menisci, subchondral bone marrow and synovium, as previously reported [16]. Tom+ cells present in the repair tissue were assessed quantitatively. Whilst joints treated with either hUC-MSCs or hBM-MSCs had more Tom+ Gdf5-lineage cells in the cartilage repair tissue than the vehicle control, this was only significant for hUC-MSCs (hUC-MSCs *p* = 0.044 and hBM-MSCs *p* = 0.752; Figure 4). The area of repair tissue, which was immuno-positive for type II collagen (mean ±95% CI), was 40.26% (±12.92) and 37.89% (±11.23) for hUC-MSCs and hBM-MSCs compared to 26.05% (±10.78) for the no cell control group, but this difference was not significant (Figure 2). Both the numbers of Tom+ cells and the area of repair tissue positively immunostained with type II collagen correlated positively with the degree of repair as assessed by Wakitani score (*p* = 0.0002 and *p* < 0.0001, respectively; Figure 2 and Figure 4).

**Figure 5:**
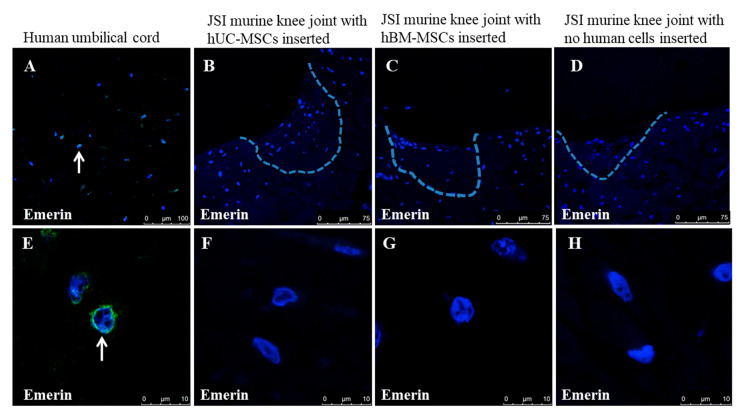
Immunostaining with the human anti-emerin antibody demonstrated (**A**,**E**) positive cells (arrows) in a section of human umbilical cord, but not in any mouse knee joints (**B**–**D**,**F**–**H**), including those which had human UC-MSCs (**B**,**F**) or human BM-MSCs (**C**,**G**) applied.

Immunostaining clearly identified human cells positively stained with the antibody to emerin in sections of paraffin-wax-embedded human umbilical cord tissue, but no human cells were detected in any of the knees from mice receiving human MSCs at the end time point (8 weeks) post-JSI (Figure 5).

### 3.2. Inflammatory Markers

**Figure A2:**
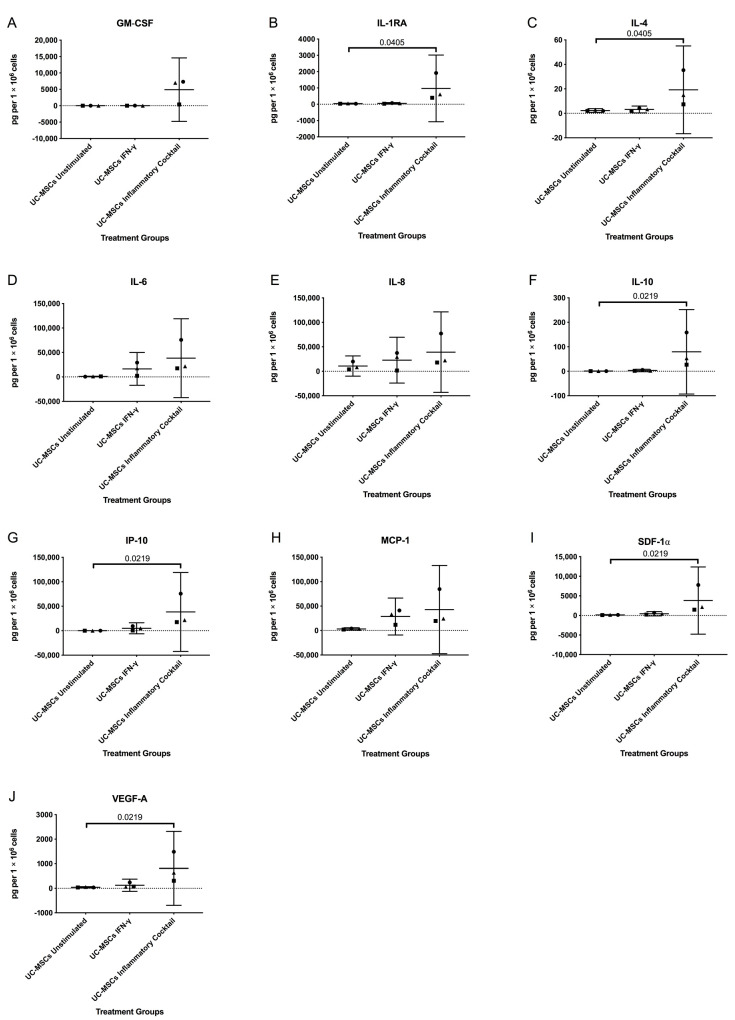
Levels of inflammatory markers measured in conditioned media collected from the following human UC-MSC cell cultures: un-stimulated, stimulated with IFN-γ (25 ng/mL for 24 h) and stimulated with an inflammatory cocktail (25 ng/mL IFN-γ, 50 ng/mL TNF-α 10 ng/mL IL-1β for 24 h) and assessed for the presence of several analytes to determine the in vitro response of the human UC-MSCs to an inflammatory environment which were applied to the injured mice knees. Stimulation with IFN-γ alone for 24 h made no significant difference from the unstimulated hUC-MSCs for any of the analytes assessed (**A**–**J**). However, IL-1RA (**B**), IL-4 (**C**), IL-10 (**F**), IP-10 (**G**), SDF-1α (**I**) and VEGF-A (**J**) were significantly increased following stimulation with an inflammatory cocktail for 24 h, compared with the unstimulated human UC-MSCs; no significant differences were observed for GM-CSF (**A**), IL-6 (**D**), IL-8 (**E**) or MCP-1 (**H**). Data are presented as the ±95% CI. Significance was determined below *p* < 0.05. ▪ = donor D1; ▲= donor D2; ● = donor D3.

The murine plasma samples from the end point of the study had undetectable levels of GM-CSF, IL-1β, IL-4, IL-6, and IFN-γ (i.e., below the lower limit of detection (LLOD)). The LLODs of these ranged between 0.12 pg/mL for IL-4 and 1.8 pg/mL for IL-6. There were only measurable quantities of IL-10, MCP-1, TNF-α, and VEGF (LLODs of 0.44, 2.63, 1.12 and 1.28 pg/mL, respectively), which showed no significant differences across the treatment groups. (There was evidence of haemolysis in some samples; however, its occurrence did not appear to significantly correlate with any of the analytes tested). In the hMSC conditioned medium, the levels of all the factors measured were increased following stimulation with the inflammatory cocktail compared to the unstimulated hUC-MSCs, being significant for IL-1RA, IL-4, IL-10, IP-10, SDF-1α and VEGF-A (Appendix A, Figure A2). Stimulation with IFN-γ alone made no significant difference from the baseline (unstimulated hUC-MSCs).

## 4. Discussion

An allogeneic cell therapy for treating joint chondral or osteochondral defects has many benefits over an autologous product in terms of logistics, and if it showed equivalent efficacy to an autologous product, would have a much greater cost–benefit than for example the currently available U.S. Food and Drug Administration (FDA)- and NICE-approved autologous chondrocyte implantation [31]. In this xenogeneic study, we have demonstrated that application of human MSCs (whether derived from bone marrow or umbilical cord) has potential as an allogeneic therapy for cartilage repair, with both cell populations resulting in better repair of a chondral injury in the murine knee than when no cells were applied.

There were a higher percentage of Tom+ Gdf5-lineage cells in the repair tissue formed in both groups of hMSC-treated mice compared to the no cell control group, although this was only significant for the hUC-MSCs. The origin and time scale of the Tom+ cells arriving in the repair tissue is unclear. As it is a reporter mouse line based on Gdf5-Cre that is active in the embryonic knee joint interzone, but not in the adult normal or OA knee [18,32,33], the Tom+ cells in the repair tissue must have derived from pre-existing Tom+ Gdf5-lineage cells that are normally found in various joint tissues including cartilage and synovium [16]. Chondrocytes could have migrated from the healthy adjacent cartilage to the repair site, as these cells have been attributed with the capability of proliferation and migration within articular cartilage in vivo [34]. In addition, progenitor cells are reported to exist in articular cartilage, particularly in the superficial zone, in bovines [35], humans [36] and mice [37]. The synovium may also act as a ‘postnatal reservoir’ of MSCs, which may contribute to maintaining joint tissues in adult life [16,21,38,39] and could perhaps have contributed to the repair tissue formation. Synovial fluid also contains cells with potential progenitor status (at least in humans) [40]. It is likely that a source of resident endogenous cells was recruited and activated by some form of paracrine signaling from the implanted MSCs, since the quality of repair tissue formed, as assessed histologically, was significantly better in these mice than those without cells.

The area of the repair tissue which was immuno-positive for type II collagen was also greater for both of the cell-treated groups compared to the no cell controls, but this difference was not significant. There were correlations between several of the histological parameters, with higher numbers of Tom+ cells in areas with a greater area of staining for type II collagen and a very highly significant correlation between the number of Tom+ cells and a decreased (i.e., better) Wakitani repair score. This implies that a greater presence of the Tom+ cells results in a better quality of repair and is consistent with our previous study that showed a correlation between the degree of cartilage repair and the presence of Gdf5-lineage cells in the repair tissue in this mouse model [16]. Certainly a greater proportion of type II collagen in repair tissue in humans following ACI is indicative of a maturation process from fibrocartilage towards hyaline cartilage, which has been shown to occur with time following cell therapy treatment [41,42].

The fact that no human cells were detectable in the repair tissue in any of the samples is not surprising considering that similar findings have been reported by others. For example, previous work found that hMSCs administered into rodent knee joints in a single injection rapidly reduced in cell number and were no longer detectable 7 days later [43]. Mancuso et al. (2019) [44] reported that unpublished data from their laboratory showed that as few as 1.6% of MSCs injected into murine OA knees were engrafted 3 days post-implantation. This may provide an indication of the mode of action (MoA) of the implanted MSCs; it is now well recognised that they produce many immunomodulatory factors and chemokines and it may be these paracrine factors, rather than their ability to differentiate into chondrocytes, that are key to their facilitating enhanced repair [45]. Indeed, whilst we find some capability of chondrogenesis by both hBM-MSCs and hUC-MSCs in vitro, Islam et al. (2016) [46] reported poor chondrogenic abilities of hUC-MSCs per se. Mancuso et al. (2019) [44] suggest that the MSCs become apoptotic and thereby modulate inflammation, being protective against OA.

Importantly for a potential allogeneic therapy, there was no evidence that implantation of these MSCs produced any immunogenic or adverse response in mice, despite being from a different species. Likewise, the synovitis score did not differ significantly between the joints from the groups of mice implanted with cells and the no cell controls, indicating the cells did not induce synovitis, at least at this time point.

Similarly, there was no evidence of any elevation of inflammatory mediators, growth factors or chemokines in the plasma of the mice when culled. However, inflammation is a transient process, and most likely to occur soon after the injury and/or implantation of the cells. Hence, only having one time point for these assessments and not being able to measure this at earlier time points is a limitation of the study. There are other studies that support our results; for example, hBM-MSCs have been transplanted into a tibial defect in sheep, with no local or systemic reactions being observed after 3 and 6 months of transplantation [47].

Whatever is the main MoA of MSCs, characterisation of individual donor populations is likely to be important in selecting donors for an allogeneic therapy. For example, if immunomodulation is the main MoA, then the level of synthesis of all of the factors as measured in the conditioned media of the different hUC-MSC populations in this study, can be seen to vary considerably between the 3 donors examined; indeed one in particular (donor 3) always resulted in higher levels of the proteins in the CM (Appendix A, Figure A2). This demonstrates how important screening of individual donors could be, with a full characterisation of pertinent assessments being defined, not only to determine their likely efficacy, but also to be used as ‘release criteria’ when up-scale manufacture is undertaken for production of the allogeneic cell therapy product.

hMSCs have already been used in knees of patients, for example hMSCs derived from bone marrow have been used and show promise in treating cartilage defects [48] and hMSCs from umbilical cord blood for treating medial knee OA [49]. However, both of these were used in conjunction with other therapeutics; the bone marrow MSCs were accompanied by applying autologous chondrons as well and the cord blood MSCs with high tibial osteotomies, such that it is difficult to know how the MSCs alone would have performed.

## 5. Conclusions

In conclusion, results from this study indicate that hUC- and hBM-MSCs have potential as a source of allogeneic cells for cell therapy to treat joint defects, although this study is limited in only involving a small animal murine model. If such results could be replicated in a large animal model, such a treatment could be transformational if applied early after an injury to slow down or stop the development of OA. This is in contrast to when the cells were applied to a severe model of degeneration, akin to more established OA [14]; when applied at this later stage of OA, the cells showed little potential for modifying or changing the disease pathway, as occurred in the present study. hUC-MSCs show advantages for up-scaled production compared to hBM-derived MSCs, due to them being easy to obtain from clinical waste and their faster proliferation rate in vitro than hBM-MSCs [10,12]. In addition, hUC-MSCs expressed mRNA for the immunomodulatory molecule, IDO (which suppresses the pro-inflammatory proliferation of T cells and NK cells) [50], at a factor of a thousand-fold-plus greater than hBM-MSCs when culture expanded in the bioreactor [12]. This may render the hUC-MSCs particularly suitable in the treatment of inflamed joints or some inflammatory arthritides, where T cells and NK cells contribute to disease progression. In addition, we have demonstrated the ability of hMSCs to promote endogenous regeneration as well as their more widely accepted immunomodulatory mode of action. Demonstrating improved cartilage regeneration should translate to longer-term health economic benefits, with a permanent improved functionality for patients rather than perhaps simply a transient anti-inflammatory effect.

## Data Availability

The data presented in this study are available on request from the corresponding author.

## Appendix A

**Table A1:** Synovitis Scoring Parameters. Parameters assessed are adapted from ^#^ Jackson et al., 2014 [22] and * Krenn et al., 2006 [23].

Score	Synovial Hyperplasia Severity ^#^	Sub-Synovial Stroma *	Sub-Synovial Inflammation ^#^	Synovial Exudate ^#^
Description	Do not score the cells actually attached to the femur. Score the maximum hyperplasia.	Score the stroma adjacent to the synovial membrane.	Score the infiltration of inflammatory cells (neutrophils, macrophages and/or lymphocytes).	Infiltration of inflammatory cells (neutrophils, macrophages and/or lymphocytes) in the synovial cavity.
0	1 cell thick	Synovial stroma shows normal cellularity.	No inflammatory cells	No inflammatory cells or fibrin in the synovial cavity.
1	Mild: 2–3 cells thick	The cellularity is slightly increased.	Occasional scattered inflammatory cells–or perivascular.	Inflammatory cells and/or fibrin colt in the synovial cavity–may be restricted to recesses.
2	Moderate: 4–5 cells thick	The cellularity is moderately increased, multinucleated cells might occur.	Focal areas of dense subsynovial WBC infiltrate–but still predominantly normal subsynovial areolar connective tissue present.	
3	Severe: >6 cells thick	The cellularity is greatly increased, multinucleated giant cells, pannus formation and rheumatoid granulomas might occur.	Widespread dense subsynovial WBC infiltrate–markedly reduced or little/no normal areolar connective tissue evident or lymphoid follicle formation.	
